# First in Man Result of Clip2Edge Transcatheter Edge-to-Edge Repair System in Heart Failure Patients: One-Year Outcomes

**DOI:** 10.1016/j.shj.2026.100804

**Published:** 2026-01-22

**Authors:** Shouzheng Wang, Wenlong Zhu, Yu Han, Weidong Li, Jing Chen, Zhenwen Yang, Zongjun Liu, Jian Yang, Xiangbin Pan, Junjie Zhang, Da Zhu

**Affiliations:** a Structural Heart Center, Fuwai Yunnan Hospital, Chinese Academy of Medical Sciences, Affiliated Cardiovascular Hospital of Kunming Medical University, Kunming, Yunnan, China; b Department of Cardiology, Fuwai Central China Cardiovascular Hospital, Zhengzhou, Henan, China; c Department of Cardiac Surgery, The First Affiliated Hospital of Zhejiang University School of Medicine, Hangzhou, Zhejiang, China; d Department of Cardiology, Renmin Hospital of Wuhan University, Wuhan, Hubei, China; e Department of Cardiology, Tianjin Medical University General Hospital, Tianjin, China; f Department of Cardiology, Putuo District Central Hospital Affiliated to Shanghai University of Traditional Chinese Medicine, Shanghai, China; g Department of Cardiac Surgery, Xijing Hospital, Xi'an, Shaanxi, China; h Department of Cardiology, Nanjing First Hospital, Nanjing, Jiangsu, China

**Keywords:** Heart failure, Mitral regurgitation, Transcatheter edge-to-edge repair

## Abstract

• Transcatheter edge-to-edge repair (TEER) has now become the standard treatment option for patients with moderate to severe or severe symptomatic functional mitral regurgitation (MR) despite the guideline-directed medical therapy.
• The MitraClip (Abbott Vascular, Santa Clara, California) is the only TEER system currently approved by the Food and Drug Administration for the treatment of functional MR.
• This study has demonstrated the initial safety and efficacy of a new TEER device (Clip2Edge system, IasoCardiac Medical Technology Co, Ltd, Shanghai, China) for treating functional MR patients who remain symptomatic despite guideline-directed medical therapy.
• This device may provide an additional treatment option for this patient population.

Transcatheter edge-to-edge repair (TEER) is the standard treatment option for functional mitral regurgitation (MR).^1^ The MitraClip (Abbott Vascular, Santa Clara, California) is the only currently available TEER system approved by the Food and Drug Administration for the treatment of functional MR. The Clip2Edge system (IasoCardiac Medical Technology Co, Ltd, Shanghai, China) is a new TEER system that incorporates a unique screw thread lock mechanism. This study aims to report the 1-year outcomes of this system in the treatment of heart failure patients with functional MR.

In this multicenter, prospective, single-arm study, symptomatic heart failure patients with 3+/4+ degree of functional MR were enrolled. The key inclusion criteria included age ≥18, symptomatic heart failure with 3+/4+ degree functional MR despite stable and maximally tolerated goal-directed medical therapy (GDMT) for at least 1 month, New York Heart Association (NYHA) functional class II-IVa, left ventricular ejection fraction ≥20% and ≤50%, and left ventricular end-systolic dimension ≤70 mm. The appropriateness of GDMT and the anatomical suitability for TEER were confirmed by an independent eligibility committee. The study protocol was developed according to the Mitral Valve Academic Research Consortium guidelines.^2^ Ethics committee approvals were obtained at each participating site. After patients signed informed consent forms, they were enrolled and underwent the TEER procedure using the Clip2Edge system. Follow-up visits were conducted at discharge, 30 days, 6 months, and 12 months after intervention. The primary endpoint was the composite rate of all-cause mortality and heart failure hospitalization (HFH) at 12 months. Echocardiograms were assessed by an independent central core laboratory. A cardiac event committee adjudicated predefined major adverse events.

The Clip2Edge system is conceptually similar to the third-generation MitraClip system. It consists of a 24F guiding catheter and a clip-delivery catheter, providing 4 clip sizes (either 4 mm or 6 mm in arm width and 9 mm or 12 mm in arm length). This system includes a unique screw thread lock mechanism to ensure sufficient locking and decrease the manufacturing difficulty (Figure 1).

**Figure 1:**
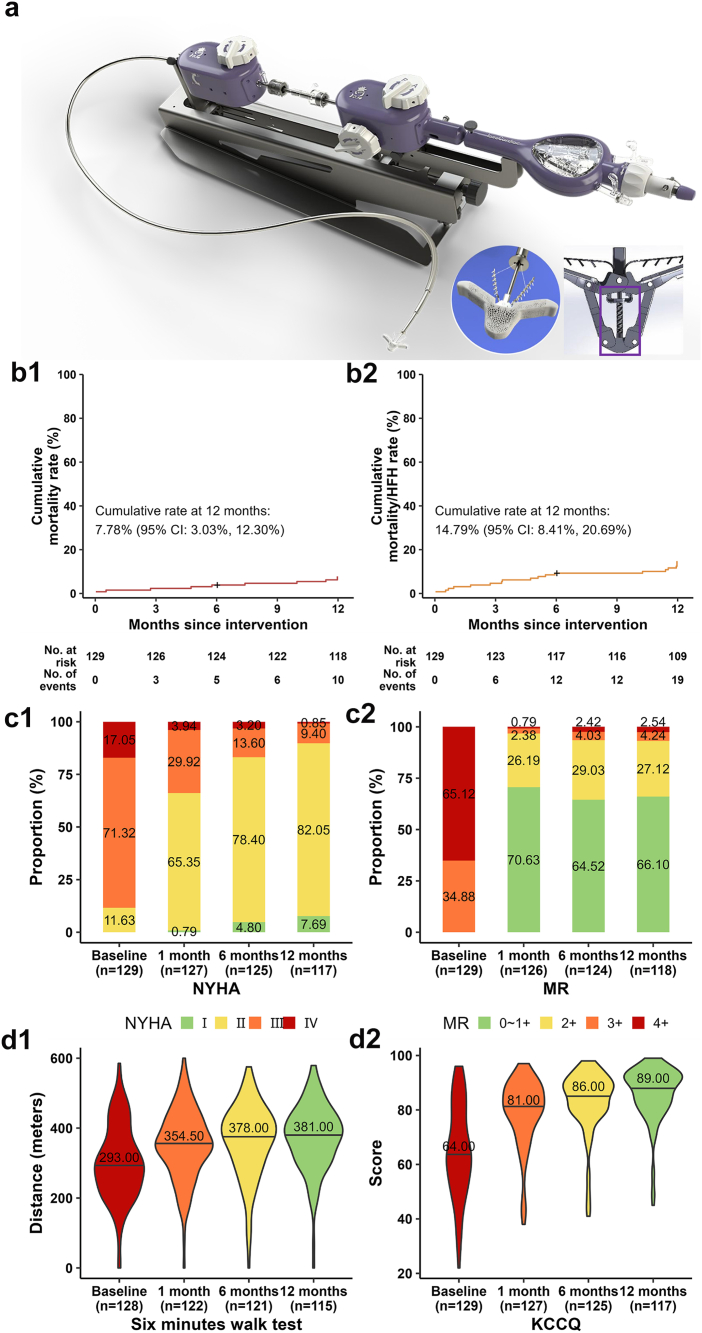
**(a)** Design of the Clip2Edge system. **(b)** Kaplan–Meier estimates for cumulative all-cause mortality rate and composite rate of all-cause mortality or HFH in 12 months follow-up. **(c)** NYHA class and MR severity from baseline to 12 months. **(d)** 6MWD and KCCQ from baseline to 12 months. Abbreviations: 6MWD, 6-minute walk distance; HFH, heart failure hospitalization; KCCQ, Kansas City Cardiomyopathy Questionnaire; MR, mitral regurgitation; NYHA, New York Heart Association.

Data are presented as numbers and percentages, means and SDs, or medians (25th percentiles [P25], 75th percentiles [P75]). Paired t-tests or paired chi-square tests were used to compare differences in 6-minute walk distance, Kansas City Cardiomyopathy Questionnaire (KCCQ) score, NYHA functional classification, degree of MR, and echocardiogram metrics at baseline and 12 months. The rates of mortality and the composite of mortality/HFH were estimated using the Kaplan–Meier method. All statistical analyses were conducted using R software (version 4.4.1, R Foundation for Statistical Computing, Vienna, Austria).

A total of 135 patients from 9 centers were enrolled, with 129 patients meeting the inclusion criteria and subsequently undergoing the TEER procedure. The mean age of the patients was 65.53 ± 8.34 years, and 56.59% (73/129) were males. Overall, 25.58% (33/129) had ischemic MR due to coronary heart disease, whereas 74.42% (96/129) presented with non-ischemic MR. Overall, 88.37% (114/129) of patients were classified as NYHA ≥ III. All patients had MR grade ≥ 3+, and 65.12% (84/129) demonstrated grade 4+ MR and the mean effective regurgitation orifice area was 0.40 ± 0.15 cm^2^. In total, 84.50% (109/129) of patients had a history of hospitalization due to heart failure within the previous 1 year. All patients received GDMT; 92.25% (119/129) received angiotensin-converting enzyme inhibitors/angiotensin receptor blockers/angiotensin receptor-neprilysin inhibitors, 90.70% (117/129) received beta-blockers, and 97.67% (126/129) received mineralocorticoid receptor antagonists, whereas 37.98% of patients (49/129) received sodium-glucose co-transporter 2 inhibitors. The mean procedure time was 102.19 ± 45.49 minutes. Single clip implantation was observed in 64.34% (83/129) of patients. Technical success was achieved in 98.45% (127/129) of patients. Major vascular bleeding was noted in 1 patient, and another patient experienced anterior leaflet perforation immediately after the procedure. At the 30-day follow-up, device success was confirmed in 94.57% (122/129) of the study cohort, with no reports of TEER device malfunction or conversion to open heart surgery.

At the 12-month follow-up, the cumulative all-cause mortality rate was 7.78% (95% CI: 3.03%, 12.30%), and the composite rate of all-cause mortality and HFH was 14.79% (95% CI: 8.41%, 20.69%). One patient was lost to follow-up at the 12-month timepoint. A total of 15 HFH events were recorded in 12 patients. Device-related major complications were noted in 3 patients, including 2 anterior leaflet perforations (both received successful transcatheter occlusion^3^) and 1 femoral vascular bleeding. No single leaflet device attachment, clip-induced significant mitral stenosis (mean mitral orifice area of 2.80 ± 0.65 cm^2^ at 12 months), device malfunction, or device-related embolism was reported during the 12 months. Other major adverse events included 4 disabling strokes (3.10%).

The site-assessed NYHA functional class showed significant improvement, with the percentage of patients categorized as class I/II rising from 11.63% at baseline to 89.74% at 12 months (*p* < 0.001). The proportion of patients with an MR grade of ≤2+ was 96.82% at 30 days, 93.55% at 6 months, and 93.22% at 12 months, respectively. KCCQ score indicated a mean improvement of Δ_mean_ = 20.68 (95% CI: 17.47, 23.89) from baseline (median: 64.00; P25, P75: 54.00, 79.00) to 12 months (median: 89.00; P25, P75: 82.00, 93.00; *p* < 0.001) along with a significant improvement of 6-minute walk distance by Δ_mean_ = 68.46 m (95% CI: 51.10, 85.82) from baseline (median: 293.00; P25, P75: 229.50, 364.00) to 12 months (median: 381.00; P25, P75: 316.00, 436.50; *p* < 0.001). A paired analysis further revealed positive remodeling of the left ventricle, evidenced by a significant reduction in left ventricular end-diastolic dimensions at 12 months (median: 58.00; P25, P75: 53.00, 68.00) compared with baseline (median: 63.00; P25, P75: 57.00, 70.00; Δ_mean_ = −3.97, 95% CI: −5.34, −2.59; *p* < 0.001) and a slight increase in ejection fraction at 12 months (median: 35.00; P25, P75: 30.00, 43.00 at baseline to median: 39.00; P25, P75: 31.25, 48.00 at 12 months; Δ_mean_ = 3.85, 95% CI: 2.35, 5.36; *p* < 0.001).

Following the encouraging results from the COAPT trial,^4^ TEER using the MitraClip system has become the standard treatment option for significant functional MR. The subsequent RESHAPE trial further broadened the indication to include patients with moderate to severe functional MR.^5^ This study presents the 12-month outcomes of a novel TEER system designed to treat patients with significant functional MR despite GDMT. The findings demonstrate reliable device performance alongside a significant improvement in NYHA functional class and KCCQ score at the 12-month follow-up. Notably, the incidence of stroke was reported at 3.1%, which is comparable to the rate of the COAPT trial (4.4%).^4^ No single leaflet device attachment, device-related embolization, or device malfunction (such as locking failure) was observed over the 12 months. In terms of efficacy, 93.22% of patients achieved an MR grade of ≤2+ at 12 months, which aligns with results from several pivotal trials using the MitraClip system.^4^^,^^5^ This study also exhibits several limitations, including its single-arm design and relatively short follow-up period, indicating that further study is necessary to validate these findings. In conclusion, this study demonstrates the initial safety and efficacy of the Clip2Edge system in treating functional MR.

## Ethics Statement

This research was carried out in accordance with the appropriate ethical guidelines. Approval from the ethics committee at each site was received, and informed consents were obtained.

## Funding

This work was supported by the Noncommunicable Chronic Diseases-National Science and Technology Major Project (2024ZD0527100), the Major Science and Technology Special Plan Project of Yunnan Province (202302AA310045), and the Yunnan Expert Workstation under the Yunnan Provincial Project for Scientific and Technological Talents and Platforms (202305AF150069).

## Disclosure Statement

The authors report no conflict of interest.
